# Immunogenicity and Protective Efficacy of Seasonal Human Live Attenuated Cold-Adapted Influenza Virus Vaccine in Pigs

**DOI:** 10.3389/fimmu.2019.02625

**Published:** 2019-11-08

**Authors:** Barbara Holzer, Sophie B. Morgan, Veronica Martini, Rajni Sharma, Becky Clark, Christopher Chiu, Francisco J. Salguero, Elma Tchilian

**Affiliations:** ^1^Enhanced Host Responses, The Pirbright Institute, Woking, United Kingdom; ^2^Department of Infectious Disease, Hammersmith Campus Imperial College London, London, United Kingdom; ^3^Public Health England, PHE Porton, Salisbury, United Kingdom

**Keywords:** LAIV (live attenuated influenza vaccine), pig, influenza virus, T cell responses, mucosal immunity, antibody responses, Fluenz® tetra, swine influenza

## Abstract

Influenza A virus infection is a global health threat to livestock and humans, causing substantial mortality and morbidity. As both pigs and humans are readily infected with influenza viruses of similar subtype, the pig is a robust and appropriate model for investigating swine and human disease. We evaluated the efficacy of the human cold-adapted 2017–2018 quadrivalent seasonal LAIV in pigs against H1N1pdm09 challenge. LAIV immunized animals showed significantly reduced viral load in nasal swabs. There was limited replication of the H1N1 component of the vaccine in the nose, a limited response to H1N1 in the lung lymph nodes and a low H1N1 serum neutralizing titer. In contrast there was better replication of the H3N2 component of the LAIV, accompanied by a stronger response to H3N2 in the tracheobronchial lymph nodes (TBLN). Our data demonstrates that a single administration of human quadrivalent LAIV shows limited replication in the nose and induces detectable responses to the H1N1 and H3N2 components. These data suggest that pigs may be a useful model for assessing LAIV against influenza A viruses.

## Introduction

Influenza virus infection is a global health threat to livestock and humans causing substantial mortality. Influenza immunization is the main strategy to control the burden of seasonal influenza, which affects 5–10% of adults and 20–30% of children, causing 650,000 deaths per year worldwide (1). The traditional intra-muscular inactivated influenza vaccine is only 50–60% effective (2) and induces only strain-specific immunity, therefore requiring repeated annual immunization to match new influenza variants. An alternative, potentially more effective, approach is the live attenuated influenza vaccine (LAIV) with absolute efficacy rates of 75–80% in children (3, 4).

LAIVs are based on the introduction of temperature sensitive (ts) and attenuating mutations in internal protein gene segments (5). Ts mutations inhibit replication of LAIV in the lungs, but these viruses are able to efficiently replicate in the lower temperatures of the nasopharynx. They are administered intra-nasally and induce a wider range of cellular, humoral and mucosal immune responses than the inactivated vaccine (6–8). The major advantage of LAIVs are the greater breadth of protection against antigenic drift variants (9, 10) as well as antigenically-shifted pandemic strains in animal models (11, 12).

LAIV is preferentially recommended for use in children, but the recommendation was withdrawn in the US because seasonal LAIV was not effective against H1N1pdm09, although no single cause for this failure was identified (13). However, recent studies showed that switching the H1N1 component might overcome the poor effectiveness reported with previous LAIV formulations (14).

Ferrets and mice are commonly used for preclinical evaluation of influenza vaccines including LAIV (11, 15–17). In mice, both cellular and humoral immunity contribute to LAIV-mediated protection, their contributions depending on the location and replication of the vaccine virus (18, 19). However, the translational relevance of the findings from the mouse model for humans are not clear because LAIVs administered intra-nasally to mice replicate in the lower respiratory tract, but do not do so in humans. In ferrets replication of LAIV is limited in the upper respiratory tract, as in humans, nevertheless there are inconsistencies between humans and these animal models (12, 20–23). African Green Monkeys were shown to be a useful animal model for evaluating vaccine efficacy, more closely resembling the human experience, but primates are expensive and their use evokes strong public opinion (24). Although the body temperature of pigs is 39°C, similar to ferrets, the pig maybe a useful alternative large animal model of human influenza infection and for LAIV testing (25, 26). Pigs are natural hosts for influenza viruses, have the same distribution of sialic acid receptors in their respiratory tract and are, physiologically and anatomically, more similar to humans than small animals (27). Experimental swine LAIVs have been tested extensively in pigs with promising results and an attenuated influenza vaccine has recently been approved for use in the USA (28, 29). LAIV carrying either an elastase cleavage site (30), non-structural protein (NS1) truncations (31), or temperature-sensitive mutations in the polymerase basic protein (PB) 2 and PB1 segments (32, 33) have been shown to be protective in pigs and LAIV consistently conferred efficient protection against matched and mismatched strains (33, 34).

However, to our knowledge the efficacy of human seasonal LAIV in pigs has never been studied. Since pigs and humans are readily infected by H1N1pdm09 viruses and the effectiveness of LAIV against H1N1pdm09 was variable, we have evaluated the immunogenicity and protective efficacy of the 2017/2018 cold adapted human quadrivalent LAIV against challenge with H1N1pdm09 in pigs.

## Materials and Methods

### Vaccine and Wild Type Viruses

The cold-adapted 2017–2018 Northern Hemisphere LAIV vaccine Fluenz Tetra (AstraZeneca) was obtained, containing two type A viruses: H1N1 A/Slovenia/2903/2015, MEDI 279432 10^7.0±0.5^ FFU [A/Michigan/45/2015 (H1N1) pdm09—like strain]; H3N2 A/New Caledonia/71/2014, MEDI 263122 10^7.0±0.5^ FFU [A/Hong Kong/4801/2014 (H3N2)—like strain] and two type B (IBV) viruses; (B/Brisbane/60/2008, MEDI 228030) 10^7.0±0.5^ FFU (B/Brisbane/60/2008—like strain) and B/Phuket/3073/2013, MEDI 254977) 10^7.0±0.5^ FFU (B/Phuket/3073/2013—like strain). The influenza A/Ann Arbor/6/60 cold-adapted (*ca*) H2N2 virus (AA *ca*) is the master donor virus of the LAIV for the A viruses and B/Ann Arbor/1/66 is the master donor virus strain for B viruses (5, 35).

For *in vitro* immunogenicity and *in vivo* challenge studies we have used the wild type (wt) viruses contained in the LAIV. The IBV B/Phuket/3073/2013 and B/Brisbane/60/2008 viruses were obtained from the Francis Crick Institute (London NW1 1AT, UK). The wt H3N2 and wt H1N1 LAIV components were obtained from the National Institute for Biological Standards and Control (NIBSC, Blanche Lane, South Mimms, Potters Bar, Hertfordshire, EN6 3QG UK): A/Michigan/45/2015 (H1N1 pmd09) and A/Hong Kong/4801/2014 (H3N2). These viruses are referred to as wt H1N1 and wt H3N2.

The exact passage history of each isolate is recorded on the data sheet provided on the NIBSC homepage. The infectious influenza viruses were provided as freeze dried allantoic fluid from embryonated SPF hen's eggs. All viruses were resuspended in sterile PBS and MDCK cells (Central Service Unit, The Pirbright Institute, UK) inoculated at recommended dilutions of the virus (10^−3^-10^−5^) to expand the viruses once, before their use for animal infection and *ex vivo* immunological assays.

The internal genes of the LAIV A viruses are from A/Ann Arbor/6/60 and are similar to the those of wt H1N1 at the protein level as follows: 79.7% for NS1, 87.7% for NS2, 83.5% for M2, 92.5% for M1, 91.6% for NP, 95.2% for PA, 96.8% for PB1, 94.1% for PB2.

For the wt H3N2 the homologies are as follows: 85.3% for NS1, 90% for NS2, 94.8% for M2, 95.2% for M1, 94.4% for NP, 96% for PA, 96.7% for PB1, 96% for PB2.

### Animals, Immunization, and Challenge Studies

All experiments were approved by the ethical review processes at the Pirbright Institute and conducted according to the UK Government Animal (Scientific Procedures) Act 1986. The Pirbright Institute conforms to ARRIVE guidelines. Eighteen 5 weeks old Landrace x Hampshire cross, female pigs were obtained from a commercial high health status herd and were screened for absence of influenza A infection by matrix gene real time RT-PCR and for antibody-free status by HI using four swine influenza virus antigens. Pigs were randomized into three experimental groups of six animals; the LAIV group was immunized once with two human doses of Fluenz Tetra, administered intra-nasally in a total of 4 ml of PBS (2 ml per nostril) using a mucosal atomisation device MAD300 (MAD, Wolfe-Tory Medical). The pigs have a much longer nasal cavity and in order to make sure that the whole nasal mucosa was exposed we used a larger dose of LAIV. The wt H1N1 group was infected intra-nasally with 6.8 × 10^6^ PFU A/Michigan/45/2015 (H1N1pmd09) per pig using the MAD300. Controls were untreated animals.

**Figure 1:**
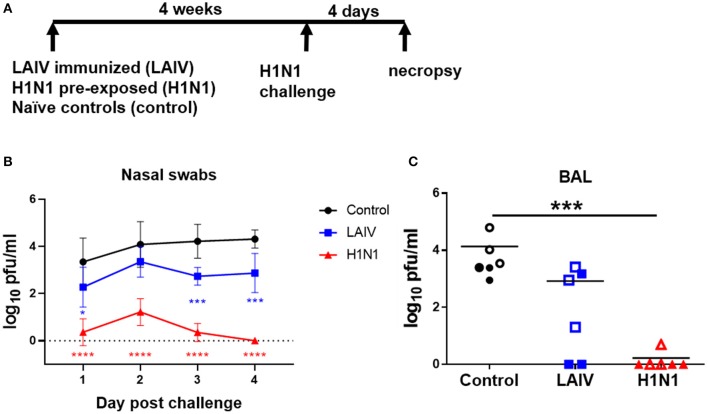
Viral load in nasal swabs and BAL. **(A)** Pigs were immunized with LAIV vaccine (LAIV immunized) or infected with wt H1N1 (H1N1 pre-exposed) or left untreated (controls). 28 or 32 days later all animals were challenged with wt H1N1 and 4 days later culled. Nasal swabs were taken at 1, 2, 3, and 4 days post challenge (DPC). Viral titers in the nasal swabs of LAIV immunized/wt H1N1 challenged, wt H1N1 pre-exposed/wt H1N1 challenged, and controls/wt H1N1 challenged and BAL at 4 DPC were determined by plaque assay. Each data point is the average of six animals challenged at either 28 or 32 days **(B)**. Animals challenged at 28 days are indicated with filled symbols and at 32 days with empty symbols **(C)**. Viral titers in the nasal swabs were analyzed using two-way ANOVA and for BAL the Kruskal–Wallis test was used. Asterisks denote significant differences ^*^p < 0.05, ***p < 0.005, ****p < 0.0005 vs. controls.

Four weeks after LAIV immunization or wt H1N1 pre-exposure all animals were challenged with A/Michigan/45/2015 (H1N1 pmd09), referred to as wt H1N1. For logistical reasons, two virus challenges were performed, with half of the animals challenged at 28 days and the remainder at 32 days post-immunization or wt H1N1 pre-exposure. The animals challenged at different times were kept in separate rooms. Animals were challenged intra-nasally as above with 6.8 × 10^6^ PFU wt H1N1 virus per pig (Figure 1A).

To assess whether wt H3N2 could infect pigs a separate group of six pigs were challenged with 9 × 10^6^ PFU wt H3N2 A/Hong Kong/4801/2014 (H3N2) per pig, intra-nasally using the MAD300.

### Pathological and Histopathological Examination of Lungs

Animals were humanely killed 4 days post challenge (dpc) with an overdose of pentobarbital sodium anesthetic. The lungs were removed and digital photographs taken of the dorsal and ventral aspects. Macroscopic pathology was scored blind as previously reported (36). Lung tissue samples were taken from the left lung and collected into 10% neutral buffered formalin for routine histological processing at the University of Surrey. Formalin fixed tissues were paraffin wax-embedded and 4 μm sections cut and routinely stained with hematoxylin and eosin (H&E). Immunohistochemical staining of influenza virus nucleoprotein was performed in 4 μm tissue sections as previously described (37). Histopathological changes in the stained lung tissue sections were scored by a veterinary pathologist blinded to the treatment group. Lung histopathology was scored using five parameters (necrosis of the bronchiolar epithelium, airway inflammation, perivascular/bronchiolar cuffing, alveolar exudates, and septal inflammation) scored on a 5-point scale of 0–4 and then summed to give a total slide score ranging from 0 to 20 and a total animal score from 0 to 100 (38).

### Tissue Sample Processing

Two nasal swabs (one per nostril) were taken daily following infection with wt H1N1 and immunization with LAIV and following subsequent challenge with wt H1N1. The swabs were placed into 2 ml of virus transport medium comprising tissue culture medium 199 (Sigma-Aldrich, UK) supplemented with 25 mM Hepes, 0.035% sodium bicarbonate, 0.5% BSA, penicillin, streptomycin and nystatin, vortexed, centrifuged to remove debris and stored at −80°C for subsequent virus titration. Serum and heparin anti-coagulated blood samples were collected at the start of the study (prior to LAIV immunization or wt H1N1 pre-exposure), before challenge and four dpc at post-mortem. Heparin blood samples were diluted 1:1 in PBS before density gradient centrifugation at 1,200 × g for 30 min over Histopaque® 1.083 g/ml (Sigma-Aldrich, UK). PBMC were harvested from the interface, washed and red blood cells lysed with Red Blood Cell Lysis Buffer (BioLegend, UK), washed again and cryopreserved in FCS (Gibco) with 10% DMSO (Sigma-Aldrich, UK). Broncho-alveolar lavage (BAL) and tracheobronchial lymph nodes (TBLN) were processed as previously described (38).

### Virus Titration and Viral RNA Detection After LAIV Administration and Challenge

Viral titers in nasal swabs and BAL fluid were determined by plaque assay on MDCK cells (Central Service Unit, The Pirbright Institute, UK). Samples were 10-fold serially diluted and 100 μl overlayed on confluent MDCK cells in 12 well tissue culture plates. After 1 h, the plates were washed and 2 ml 1:3 2% agarose:medium overlayed. Plates were incubated at 37°C for 48–72 h and plaques visualized using 0.1% crystal violet.

Viral RNA was extracted from nasal swab samples post immunization or infection with the MagVet™ Universal Isolation Kit (LSI, Laboratory Service International) according to the manufacturer's instructions on the MagMax™ Express 96 automatic extraction platform (Applied Biosystems). Samples were quantified by Reverse Transcription quantitative Polymerase Chain Reaction (RT-qPCR) (Quantitect Probe RT PCR Kit, Qiagen, UK) for the influenza A virus HA gene using an MxPro 3500P instrument and MxPro analysis software (Agilent). Briefly, RT was performed at 50°C for 30 min followed by 15 min at 95°C; 2-step cycling was then performed with denaturation for 15 s at 94°C combined with annealing/extension collection (60 s at 60°C) and this was repeated for 40 cycles. The HA-specific and subtype specific forward and reverse oligos as well as the probe sequences for the Taqman assays are available upon request. The sequences used for the design of the subtype specific Taqman assays were retrieved from public databases (GISAID or NCBI). The RNA quantity was expressed as relative equivalent units (REU) of RNA using a standard 10-fold dilution series of RNA purified from the LAIV vaccine with known LAIV titer (FFU/ml) of each of the components. RNA was extracted from duplicate nasal swab samples from each time point and quantified by RT-qPCR. Only samples where all replicates had a CT value of <40 were considered as positive for the respective LAIV component or wild type virus.

### Microneutralization Assay

Neutralizing Ab titers were determined in serum and BAL fluid using a microneutralization (MN) assay as previously described (39, 40). In brief, pig sera were heat-treated for 30 min at 56°C and 1 in 10 diluted as starting point for the assay. The serially diluted sera were incubated with an equal volume of 50 μl of virus (each virus was titered beforehand in the absence of serum to determine the PFU/ml necessary to yield a plateau infection in the MN assay). After 2 h MDCK SIAT-1 cells at 3 × 10^4^ cells/well were added to the serum/virus and incubated for 18 h. The fixed and permeabilized cell monolayer was stained with anti-nucleoprotein (Clone: AA5H, Bio-Rad Antibodies, UK) followed by goat anti mouse HRP (DAKO) antibody. After addition of the TMB substrate the reaction was stopped with 1 M sulfuric acid and absorbance was measured at 450 and 570 nm (reference wavelength) on the Cytation3 Imaging Reader (Biotek). The MN titers were expressed as half maximal inhibitory dilution (50% Inhibitory titer is: midpoint between uninfected control wells and virus-infected positive controls) derived by linear interpolation from neighboring points in the titration curve. As positive control for the MN assay we used reference sera raised in sheep provided by the National Institute for Biological Standards and Control (NIBSC, Blanche Lane, South Mimms, Potters Bar, Hertfordshire, EN6 3QG, UK): Influenza anti-A/Michigan/45/2015-like HA serum (NIBSC code: 16/304), Influenza anti-A/Hong Kong/4801/2014-like HA serum (NIBSC code: 16/182), Influenza anti-B/Phuket/3073/2013 HA serum (NIBSC code: 15/150), and Influenza anti-B/Brisbane/60/2008-HA serum (NIBSC code: 15/312).

### Enzyme-Linked Immunosorbent Assay (ELISA)

Antibody titers against the A/Michigan/45/2015 HA in the BAL fluid were determined by ELISA. The recombinant protein was expressed in HEK293 cells with a C-terminal His-tag (Stratech, UK, Cat.No. 40567-H08H-SIB). Ninety six-well microtiter plates (Maxi Sorp, Nunc, Sigma-Aldrich, UK) were coated with 50 μL recombinant protein at a concentration of 2 μg/mL in carbonate buffer overnight at 4°C. The next day, 200 μL blocking solution (phosphate-buffered saline (PBS; Central Service Unit, The Pirbright Institute, UK) supplemented with 0.05% Tween-20 (T-PBS), 4% milk powder were added to all wells of the microtiter plates and incubated for 2 h at room temperature. BALF samples were sterile filtered with a 0.22 μM tip filter and were diluted to a starting concentration of 1 in 20 in blocking buffer, and serially diluted 1:2, and incubated for 2 h at room temperature on a rocking platform. The microtiter plates were washed three times with T-PBS and 100 μL anti-pig IgG HRP or anti-pig IgA HRP antibody (Bio-Rad Antibodies, UK) diluted 1:20,000 in blocking solution was added to all wells and incubated for 2 h at room temperature on a rocking platform. The microtiter 96-well plates were washed four times with T-PBS and were developed with 100 μL/well TMB High Sensitivity substrate solution (BioLegend, UK). After 5–10 min the reaction was stopped with 100 μL 1 M sulfuric acid and the plates were read at 450 and 570 nm with the Cytation3 Imaging Reader (Biotek). The data were analyzed in Microsoft Excel and GraphPad Prism. The cutoff value was defined as the average of all blank wells plus three times the standard deviation of the blank wells. Endpoint titers were expressed as the highest dilution of the respective sample equal or above the cut-off value.

### IFNγ ELISpot

Frequencies of IFNγ-secreting cells in cryopreserved PBMC, BAL, and TBLN cells were determined by ELISpot. MultiScreen™-HA ELISpot plates (Merck, Millipore, UK), were coated with 0.5 μg/ml of anti-pig IFNγ, clone P2G10 (BD Pharmingen, UK) in carbonate buffer and incubated at 4°C overnight. The plates were washed five times in PBS and blocked using complete RPMI. After five washes in PBS, 2.5 × 10^5^ cells were seeded in triplicate wells and stimulated with either live MDCK-grown wt H1N1 or wt H3N2 (MOI = 1), medium control or 10 μg/ml Concanavalin A (Sigma-Aldrich, UK). Plates were incubated for 36 h at 37°C in a 5% CO_2_ incubator, followed by washes with PBS, 0.05% Tween20 and addition of 0.25 μg/ml anti-pig biotinylated IFNγ detection Ab, clone P2C11 (BD Pharmingen, UK). Plates were incubated for 2 h at room temperature, washed five times and streptavidin alkaline phosphatase (Invitrogen, UK) was added for a further 1 h at room temperature. Spots were visualized using alkaline phosphatase substrate kit (Bio-Rad, UK) and the reaction was stopped using tap water. Immunospots were counted using the Immunospot ELISPOT analyzer (C.T.L). Results were expressed as number of IFNγ-producing cells per 10^6^ cells after subtraction of the average number of IFNγ-secreting cells in medium control wells.

### Flow Cytometry

Cryopreserved mononuclear cells from blood, TBLN and BAL were thawed and stimulated for 12 h at 37°C with live MDCK-grown wt H1N1 or wt H3N2 (MOI = 1) or media control. GolgiPlug (BD Biosciences, UK) was added according to the manufacturer's instructions for a further 5 h before intracellular cytokine staining (ICS). Cells were stained for surface markers with CD3ε-PeCy7 BB23-8E6-8C8, CD4 74-12-4 PerCpCy5.5, CD8α-FITC 76-2-11 (BD Biosciences, UK) and Near-Infrared Fixable Live/Dead stain (Invitrogen, UK). Cells were fixed and permeabilized using Cytofix Cytoperm (BD Biosciences, UK) before intracellular staining with IFNγ AF647 P2G10 (BD Biosciences, UK) and cross-reactive anti-human TNFα-BV650 Mab11 (BioLegend, UK). Samples were fixed in 1% paraformaldehyde before analysis using an LSRFortessa (BD Biosciences). Data was analyzed by Boolean gating using FlowJo v10 (Treestar).

### Statistical Analysis

Kruskal–Wallis test or two-way ANOVA with Dunnets post-test for multiple comparisons in GraphPad Prism (version 8.0.1) were used to analyze the experimental groups.

### Data Availability

All data generated or analyzed during this study are included in this published article.

## Results

### Viral Load and Lung Pathology

To test the efficacy of human seasonal LAIV against wt H1N1 challenge, groups of six pigs were immunized with commercial LAIV Fluenz Tetra intra-nasally. As a positive control for the challenge, a group of pigs were infected intra-nasally with wt H1N1 (pre-exposed wt H1N1 group) (Figure 1A). The third group were untreated controls. Four weeks after the LAIV immunization or wt H1N1 pre-exposure, all animals were challenged with wt H1N1 virus and culled 4 days post challenge (dpc).

Viral load was assessed in nasal swabs. The wt H1N1 pre-exposed pigs showed the greatest and statistically significant reduction of challenge virus in the nasal swabs at all time points (Figure 1B). LAIV reduced viral shedding in nasal swabs, which reached significance 1, 3, and 4 dpc. No virus was detected in the BAL of the wt H1N1 pre-exposed group. LAIV reduced viral load in the BAL (*p* = 0.11) and no virus was detected in two animals (Figure 1C).

**Figure 2:**
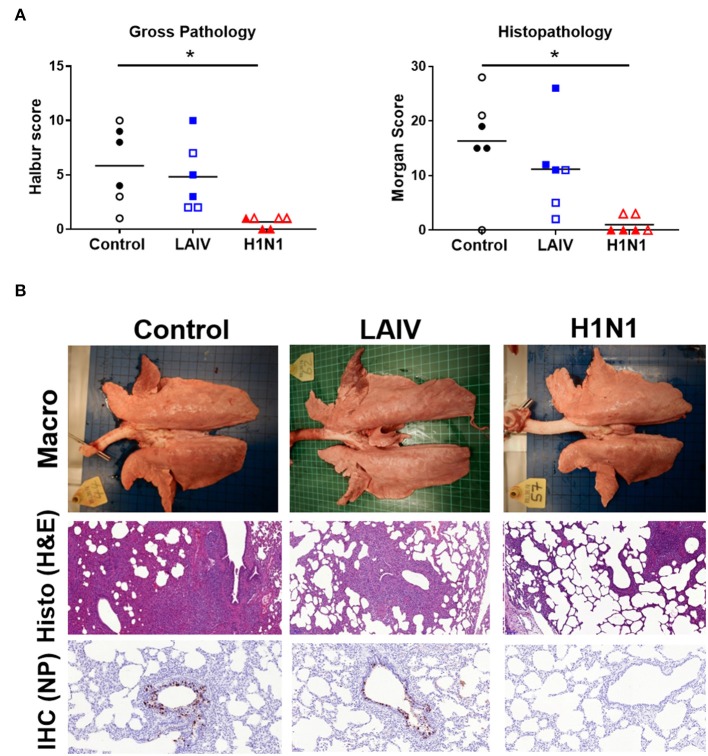
Lung pathology. Pigs were immunized with LAIV or infected with wt H1N1. Controls were untreated animals. Twenty-eight (filled symbols) or thirty-two (empty symbols) days later all animals were challenged with wt H1N1 and 4 days later culled. **(A)** Lung lesions at post-mortem were assessed macroscopically and microscopically. **(B)** Representative gross pathology, histopathology (H&E staining; 100x) and immunohistochemical NP staining (200x) of representative sections for each group are shown. Asterisks denote significant differences between the indicated groups. *p < 0.05 as analyzed by Kruskal–Wallis test. As the analysis of samples from pigs challenged at days 28 and 30 did not show any significant differences, for simplicity in presentation the results of the assays carried out on pigs challenged on both days have been amalgamated in this and the following figures.

Following wt H1N1 challenge the unimmunized control animals developed typical gross lesions of influenza virus infection, consisting of areas of broncho-interstitial pneumonia, mainly in the apical and medial lobes (41). Histopathology showed lesions consisting of multifocal interstitial pneumonia, attenuation of the bronchial and bronchiolar epithelium, inflammatory infiltrates within the interalveolar septa and the alveolar lumen, and oedema. Immunohistochemical detection of influenza virus nucleoprotein (NP) showed many positive cells within the epithelium of bronchi and bronchioles (Figure 2). In contrast the wt H1N1 pre-exposed animals showed very few gross pathological lesions. Histologically, only a few lung sections showed mild interstitial pneumonia and necrosis of the bronchial and bronchiolar epithelium. Virus NP immunostaining was restricted to very few inflammatory cells within the inter-alveolar septa. LAIV immunized animals showed moderate gross pathology and histopathology with presence of abundant immunostained bronchial and bronchiolar epithelial cells (Figure 2B).

These results indicated that immunization of pigs with human seasonal LAIV significantly reduced the viral load in nasal swabs 1, 3, and 4 dpc, against challenge with wt H1N1.

### Vaccine and Virus Shedding

**Figure 3:**
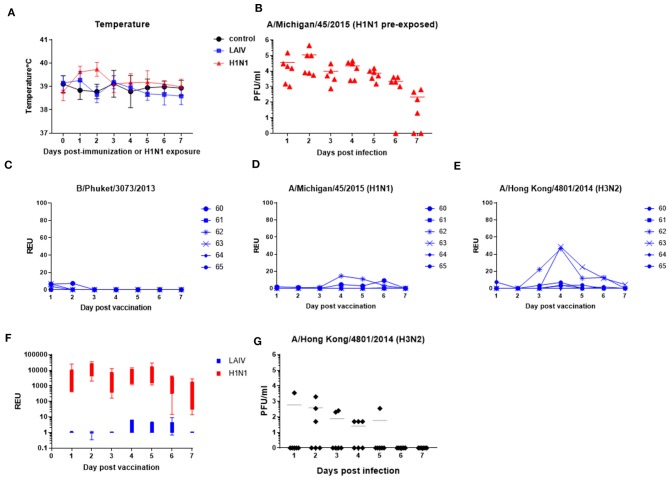
Vaccine and wt virus replication in nasal swabs. Pigs were immunized with LAIV or infected with wt H1N1. Control were untreated animals. Nasal swabs and temperatures were taken daily. **(A)** Temperatures after LAIV immunization or wt H1N1 pre-exposure. **(B,G)** Virus shedding in nasal swabs in wt H1N1-or wt H3N2 pre-exposed group determined by plaque assays. **(C–E)** RT-qPCR for the indicated virus components in the LAIV immunized animals. Each data point represents an individual within the indicated group. **(F)** Comparison of H1N1 virus shedding in LAIV immunized and wt H1N1 pre-exposed groups as determined by relative copies of H1 HA RNA. RNA quantity is expressed as Relative equivalent units (REU).

After LAIV immunization or wt H1N1 pre-exposure daily temperature and nasal swabs were taken for 7 days. There was slight elevation of temperature in the wt H1N1 pre-exposed group, but not in the LAIV immunized group (Figure 3A). No other clinical signs were observed. Vaccine and virus replication (pre-exposure group) was determined in nasal swabs. Live wt H1N1 virus was detected in nasal swabs in the wt H1N1 pre-exposed animals by plaque assay (Figure 3B). All animals were infected and virus shedding peaked at day 2 post pre-exposure. However, in the LAIV group virus was detected only by RT-qPCR in order to discriminate between the four different viral LAIV components. Thus, separate RT-qPCRs with HA subtype-specific Taqman probes were performed. A very low quantity of B/Phuket/ 3073/2013 virus was detected in three animals only in the first 2 days (Figure 3C) and no replication of B/Brisbane/60/2008 (data not shown). Replication of H1N1 on more than one day was detected in two out of six animals (33%) and H3N2 in four out of six (66%) (Figures 3D,E). Replication of both viruses was seen in two out of the six animals. There was no correlation between the level of protection against wt H1N1 challenge and replication of the vaccine in the nasal passage.

Relative copy numbers of H1 hemagglutinin (HA) RNA in the wt H1N1 pre-exposed group was determined by RT-qPCR in order to directly compare them to the LAIV immunized group (Figure 3F). On average there were 3 log less copies in the LAIV compared to the wt H1N1 pre-exposed group. The LAIV was administered at 2 × 10^7±0.5^ FFU/ml per pig whereas the wt H1N1 inoculum was 6.8 × 10^6^ PFU/ml per pig.

Because there was a suggestion that more H3N2 than H1N1 could be detected in the LAIV group, we next assessed whether pigs could be infected with wt H3N2. Six pigs were intra-nasally inoculated with the virus using MAD. Only four out of the six pigs shed virus (Figure 3G), with one shedding virus only on 1 day, in contrast to the six out of six infected by wt H1N1 (Figure 3B), indicating that wt H1N1 more readily infects pigs, while A/Hong Kong/4801/2014 (wt H3N2) might require additional adaptations (42). Because half of the animals were not infected by wt H3N2, we chose not to challenge LAIV immunized animals with wt H3N2.

In summary LAIV H1N1 replication in the nose of pigs was severely restricted and detected in only two out of six animals, while LAIV H3N2 replication was detected in four out of six animals. Although the H3N2 LAIV component replicated better than H1N1 in the nose, all pigs were readily infected with the wt H1N1, shedding up to 10^6^ pfu/ml, whereas infection with wt H3N2 resulted in only half of the animals being infected with a lower titer of shedding.

### Antibody and IFNγ ELISpot Responses in Pigs

**Figure 4:**
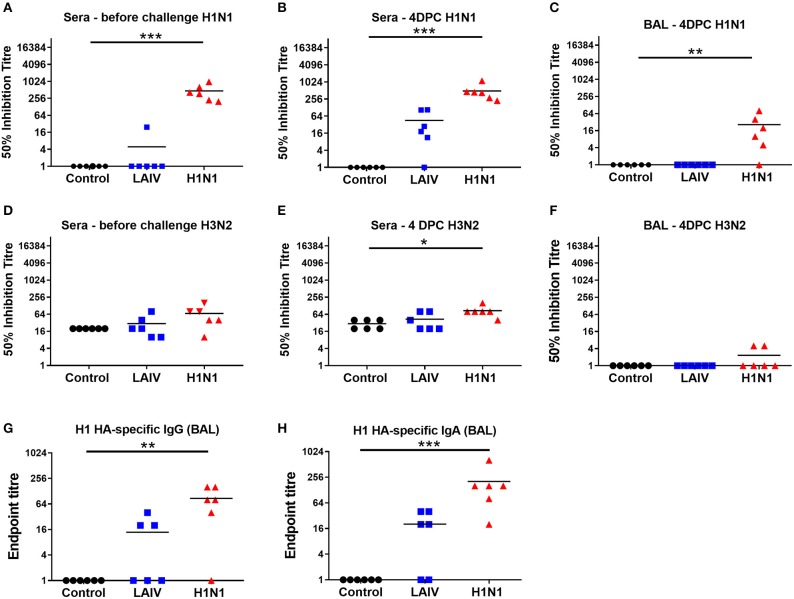
Antibody responses in pigs. Pigs were immunized with LAIV or infected with wt H1N1. Controls were unimmunized animals. All animals were challenged with wt H1N1 4 weeks after LAIV immunization or wt H1N1 pre-exposure and culled 4 days later. Fifty percentage neutralization titers against wt H1N1 and wt H3N2 in the serum collected before challenge and post LAIV immunization or wt H1N1 pre-exposure **(A,D)**, 4 days post challenge (DPC) **(B,E)**, or in the BALF at 4 DPC **(C,F)** were calculated as linear interpolation of neighboring points. H1 HA-specific IgG **(G)** and IgA **(H)** antibodies were determined by endpoint ELISA. Kruskal–Wallis test was used for the statistical analysis of neutralizing titer in the BAL and sera as well as for the ELISA endpoint titers. Asterisks denote significant differences *p < 0.05, **p < 0.05, ***p < 0.005 vs. controls.

We determined the antibody response in pigs using microneutralization assay. Sera from the wt H1N1 pre-exposed group had neutralizing antibody with 50% mean wt H1N1 inhibitory titers of 1:470 at 4 weeks after the first exposure, just before the second challenge (Figure 4A). There was a weak boosting of the Ab response after the second challenge with the mean serum titer of 1:490 at 4 dpc (Figure 4B), most likely because 4 dpc is too early to the peak of the secondary response. There was a minimal serum Ab response in the LAIV group of 1:45 at 4 dpc (Figure 4B). A titer of 1:26 was detected in the BAL of wt H1N1 pre-exposed group (Figure 4C). No neutralizing antibody to wt H1N1 was detected in the unimmunized controls. Neutralizing antibody with a 50% inhibitory wt H3N2 titer of 1:80 was detected in the serum of the wt H1N1 pre-exposed group and 1: 40 in the LAIV group (Figures 4D,E). No neutralizing response against wt H3N2 was detected in the BAL of both groups (Figure 4F). In order to analyze the mucosal antibody responses induced by LAIV immunization or wt H1N1 pre-exposure we performed ELISA to detect H1 HA-specific IgA and IgG antibodies in the BAL (Figures 4G,H). In the wt H1N1 pre-exposed animals average titers for IgG and IgA were >80 and 200, respectively. A proportion of the animals from the LAIV group had low titers for HA-specific IgA and IgG antibodies in the BAL. No H1 HA-specific antibodies were detected in the unimmunized animals. This suggests that non-neutralizing antibodies in the BAL of the LAIV animals might have contributed to the observed reduction in viral load. Virus specific IgA in nasal secretions has been shown to be important for virus clearance (43). However, we were unable obtain reproducible and convincing results for virus specific IgA antibodies in the nasal swabs, perhaps because of interference by virus present at 4 dpc.

**Figure 5:**
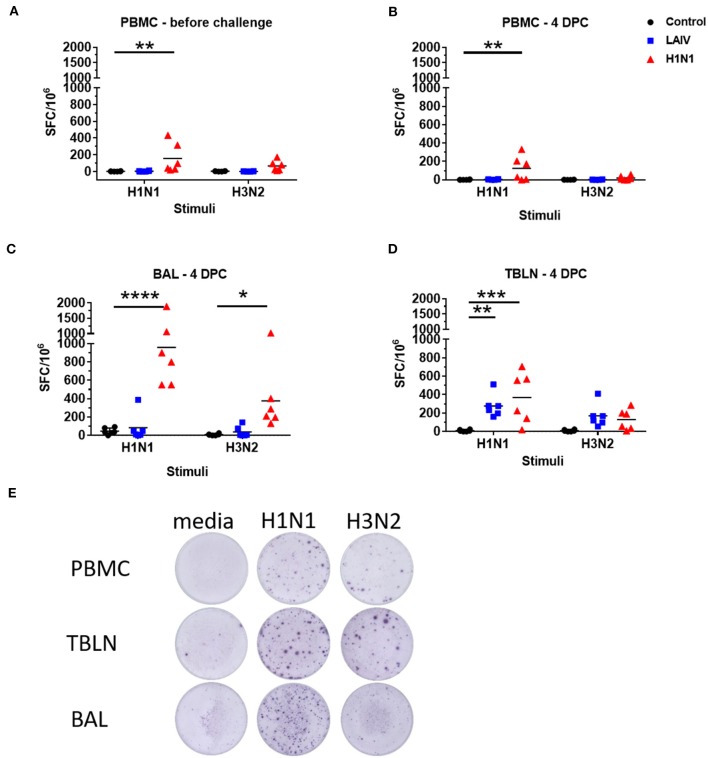
Numbers of IFNγ spot forming cells (SFC) in PBMC **(A,B)**, BAL **(C)**, and tracheo-bronchial lymph node (TBLN) **(D)** were determined by ELISpot following stimulation with wt H1N1 or wt H3N2 viruses *in vitro*. Results for wt H1N1 and wt H3N2 stimulation were expressed as number of IFNγ-producing SFC per 10^6^ cells after subtraction of the average number of IFNγ-secreting cells in medium control wells. Representative images of the ELISPOT wells are shown in **(E)**. Asterisks denote significant differences between the indicated groups *p < 0.05, **p < 0.05, ***p < 0.005, ****p < 0.0005 determined using two-way ANOVA with Dunnet's test for multiple comparisons.

We determined influenza-specific T cell responses in PBMC in pigs by IFNγ ELISpot just before the challenge (4 weeks post LAIV immunization or wt H1N1 pre-exposure), and at the time of necropsy 4 dpc (Figures 5A,B). PBMC were stimulated with either the wt H1N1, wt H3N2 or Brisbane and Phuket B viruses. The wt H1N1 pre-exposed group showed a virus specific response to both wt H1N1 and wt H3N2 viruses at the time of challenge (4 weeks after the first exposure). The response was higher following wt H1N1 *ex vivo* stimulation compared to wt H3N2 (mean 141 SFC per 10^6^ cells to wt H1N1 and 67 SFC to wt H3N2). After wt H1N1 re-challenge the response was not boosted in the pre-exposed wt H1N1 group and remained the same for wt H1N1 (123 SFC per 10^6^ cells) and was much reduced for wt H3N2 (19 SFC per 10^6^ cells). No detectable response was observed to wt H1N1 or wt H3N2 *ex vivo* stimulation in PBMC of the LAIV/ challenged group. No responses were detected to B viruses (data not shown).

IFNγ-secreting BAL cells in the wt H1N1 pre-exposed group showed a similar trend but their frequencies were significantly higher (958 SFC per 10^6^ cells for wt H1N1 and 374 SFC for wt H3N2) (Figure 5C). There was no detectable response in the BAL of LAIV immunized animals, except for one animal with 386 SFC per 10^6^ cells for wt H1N1 and 141 SFC for wt H3N2. However, there was a strong IFNγ ELISPOT response in the TBLN for the LAIV and wt H1N1 pre-exposed groups to *ex vivo* stimulation with both wt H1N1 and wt H3N2 viruses (Figures 5D,E). The response to wt H3N2 stimulation was similar between the LAIV and wt H1N1 pre-exposed group (168 SFC per 10^6^ cells for the LAIV and 128 SFC for the wt H1N1 groups), while pre-exposed animals showed a higher proportion of wt H1N1 virus specific cells (368 SFC) than LAIV (276 SFC) in the TBLN.

These data show that as expected pre-exposure to wt H1N1 induced a strong antibody response, while in contrast LAIV induced little neutralizing antibody in serum. There was a strong wt H1N1 IFNγ response after wt H1N1 pre-exposure and re-challenge in blood, BAL and TBLN. IFNγ secreting cells were detected in TBLN after LAIV immunization.

### Analysis of Cytokine Producing Cells

In order to dissect which cells produce cytokines, we performed intracellular cytokine staining for IFNγ and TNF combined with surface staining for CD4+, CD8α high, and CD4+CD8α+ cell subsets, the latter being antigen experienced CD4+ T cells in pigs (44). To analyze local immune responses TBLN and BAL cells were stimulated with either the wt H1N1 or wt H3N2, as there had been no detectable responses to the B viruses by ELISpot.

**Figure 6:**
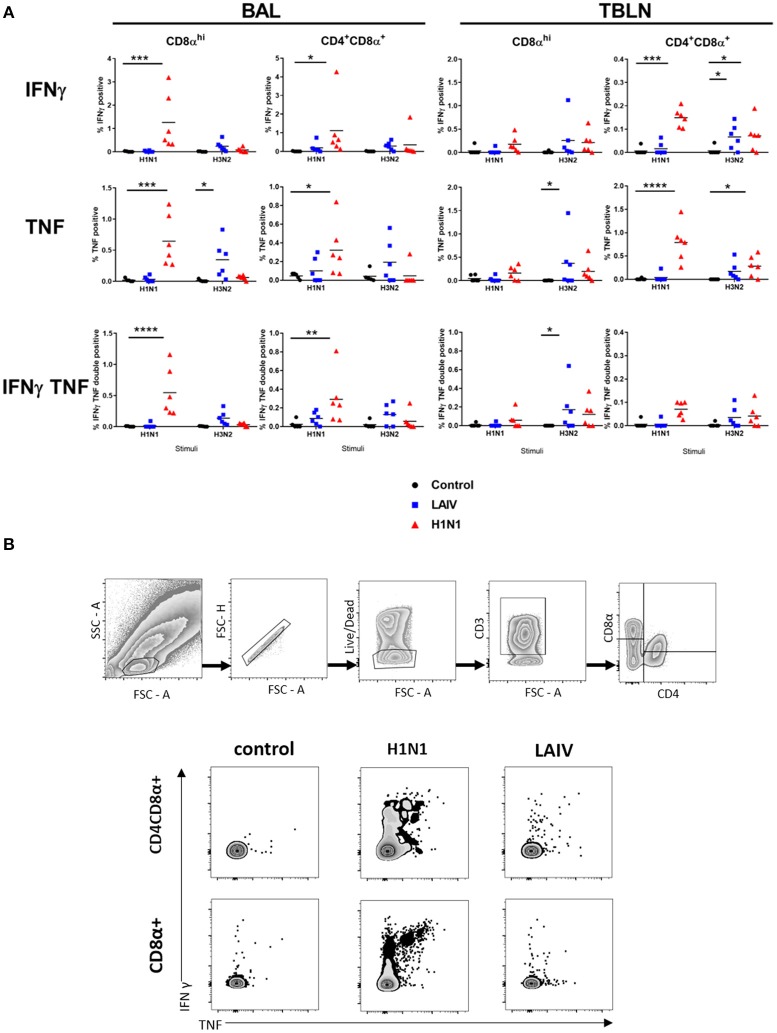
Cytokine producing cells in TBLN and BAL. Pigs were immunized with LAIV or infected with wt H1N1. Controls were untreated. All animals were challenged with wt H1N1 4 weeks after immunization or wt H1N1 pre-exposure and culled 4 days later. Lymphocytes isolated from TBLN and BAL at day 4 post challenge were *in vitro* stimulated with wt H1N1 or wt H3N2. Flow cytometry was used to quantitate the frequency of IFNγ, IFNγTNF, and TNF positive cells within CD8α^hi^ and CD4CD8α cells in BAL and TBLN. The media only has been subtracted. Each data point represents an individual within the indicated group, and bars represent the mean **(A)**. Gating strategy and representative FACS plots of BAL cells are shown in **(B)**. Data were analyzed using two-way ANOVA with Dunnet's test for multiple comparisons. Asterisks denote significant differences between the indicated groups *p < 0.05, **p < 0.05, ***p < 0.005, ****p < 0.0005.

Wt H1N1 pre-exposure induced the highest proportion of single IFNγ, single TNF and double IFNγ+TNF+ cytokine producing CD8α and CD4+CD8α+ cells in the BAL to wt H1N1 *ex vivo* stimulation (Figure 6) and minimal response to wt H3N2. There was a good response in TBLN to both wt H1N1 and wt H3N2 stimulation only in the CD4CD8α cell subset (as IFN and TNF single cytokine secreting cells) (Figure 6). Interestingly the LAIV immunized group induced a statistically significant wt H3N2 response in the TBLN (CD8α TNF and CD8α IFNγTNF and CD4CD8α IFNγ) and a TNF response in the CD8α cells in the BAL. The discrepancy with the ELISpot data may be because the ELISpot does not detect TNF production and that other cells secreting INFγ, for example NK or gamma delta cells, as well as CD4 and CD8 T cells may be detected.

Overall the data show that wt H1N1 pre-exposure induces a pronounced local response in BAL and TBLN, detected by *ex vivo* stimulation with wt H1N1 virus, while LAIV immunization generally induces a much stronger response to wt H3N2 than wt H1N1.

## Discussion

We evaluated the efficacy of human quadrivalent seasonal LAIV in pigs against wt H1N1 challenge. The LAIV immunized animals showed significantly reduced viral load in nasal swabs at day 1, 3, and 4 post challenge and although there was reduction in BAL viral load and pathology these did not reach statistical significance. There was limited replication of the H1N1 component of the vaccine in the nose, a limited response to wt H1N1 in the TBLN and a low neutralizing wt H1N1 serum titer. From this is not possible to determine the mechanism that led to a reduction in viral load in LAIV immunized animals. In contrast there was better replication of the H3N2 component in the LAIV group, accompanied by a strong response to wt H3N2 in the TBLN, but unfortunately we were unable to assess if this was protective, because a separate experiment indicated that wt H3N2 only infects a proportion of pigs.

The pig model has some limitations. The first is that because pigs are not easily infected with influenza B viruses, the efficacy of vaccines against B viruses cannot be tested. The lack of replication or immune responses to influenza B viruses in the LAIV immunized pigs confirms this. We also show that pigs were not easily infected with the human wt H3N2 virus intra-nasally, although perhaps intra-tracheal or aerosol challenge might have been more effective. However, some influenza viruses, including H1N1pdm09, infect both pigs and humans equally well. In the light of the failure to infect animals with wt H3N2, it is surprising that there is such good replication and response to the H3N2 component of LAIV. However, this may be because the internal genes of wt H3N2 are different from those of the temperature sensitive LAIV H3N2. In general it appears that the extent of replication of the LAIV viruses correlates with the magnitude of the immune response H3N2 > H1N1 > B. This is in agreement with the finding that the H3N2 component of LAIV is a better immunogen than H1N1 in humans (45).

Interestingly LAIV induced a response mainly in the TBLN, except for a TNF producing component in the BAL as detected by ICS. We speculate that this might be because after LAIV immunization with minimal replication of viral components in the lung, only a small amount of antigen reaches the TBLN to stimulate responding cells. There is insufficient antigen remaining in the lung tissues and minimal inflammation, so that most of the cells in the node do not recirculate and home to the lung after vaccination. Following wt H1N1 challenge, antigen again reaches the TBLN and induces proliferation of these memory cells, but at 4 dpc these did not have time to recirculate and home to the lung. Since the animals were immunized with a nasal spray, more antigen was deposited in the nasal cavity and it would be interesting in a future experiment to compare immune responses in the nodes draining the nasal cavity with nodes draining the lung tissues and similarly responses in the nasal mucosa with those in the BAL and lung tissue.

We have administered LAIV vaccine only once to influenza naïve pigs. Furthermore, the LAIV we used contained 4 components which might compete. In most animal models a single cold adapted virus and more often two doses of LAIV are given (15, 16, 46). In naïve infants two doses of LAIV are used. Perhaps priming and boosting is required to elicit the full effect of LAIV, which may induce a stronger mucosal response. Studies by Subbarao et al. showed that priming with LAIV followed by inactivated vaccine induced a rapid and robust antibody responses, suggesting that LAIV can establish a long-lasting memory which can be recalled with a single dose of inactivated vaccine (23, 47, 48). It would be interesting to replicate these experiments in pigs, although in this study we established the baseline effect of a single dose of LAIV.

Our data demonstrates that a single administration of human quadrivalent LAIV shows limited replication in the nose and induces detectable responses to the H1N1 and H3N2 components. LAIV immunized pigs showed reduced viral load in nasal swabs following wt H1N1 challenge. These data suggest that pigs may be a useful model for assessing LAIV against influenza A viruses and investigating mechanisms determining protective efficacy.

## Data Availability Statement

All datasets generated for this study are included in the article/supplementary material.

## Ethics Statement

All experiments were approved by the ethical review processes at the Pirbright Institute (AWERB) and conducted according to the UK Government Animal (Scientific Procedures) Act 1986. The Pirbright Institute conforms to ARRIVE guidelines.

## Author Contributions

ET, SM, BH, and VM designed the experiments. ET, BH, SM, VM, RS, BC, and FS performed the experiments and analyzed the data. BH, SM, VM, and FS prepared the figures for publication. ET wrote the paper. ET, CC, FS, BH, SM, and VM reviewed, critiqued, and provided comments on the text. CC provided reagents and advice on experimental design.

### Conflict of Interest

The authors declare that the research was conducted in the absence of any commercial or financial relationships that could be construed as a potential conflict of interest.
